# Variations in cervico-vaginal microbiota among HPV-positive and HPV-negative asymptomatic women in Peru

**DOI:** 10.1186/s13104-020-05422-6

**Published:** 2021-01-06

**Authors:** Hugo Carrillo-Ng, Lorena Becerra-Goicochea, Yordi Tarazona-Castro, Luis Pinillos-Vilca, Luis J. del Valle, Miguel Angel Aguilar-Luis, Carmen Tinco-Valdez, Wilmer Silva-Caso, Johanna Martins-Luna, Isaac Peña-Tuesta, Ronald Aquino-Ortega, Juana del Valle-Mendoza

**Affiliations:** 1 School of Medicine, Research and Innovation Center of the Faculty of Health Sciences, Universidad Peruana de Ciencias Aplicadas, Av. San Marcos Cuadra 2, Chorrillos, Lima, Peru; 2 Laboratorio de Biologia Molecular, Instituto de Investigación Nutricional, Lima, Peru; 3 Hospital Regional Docente de Cajamarca, Cajamarca, Peru; 4 Escuela Profesional de Genética y Biotecnología, Facultad de Ciencias Biológicas, Universidad Nacional Mayor de San Marcos, Lima, Peru; 5 Departament d’Enginyeria Química, EEBE, Barcelona Research Center for Multiscale Science and Engineering, Universitat Politècnica de Catalunya (UPC), Barcelona, Spain

**Keywords:** Peru, HPV, Human papillomavirus, Cervical cancer, Microbiota, PCR

## Abstract

**Objective:**

To characterize the cervicovaginal microbiota of HPV-positive and HPV-negative asymptomatic Peruvian women, by identifying the presence of 13 representative bacteria genus.

**Results:**

A total of 100 HPV-positive and 100 HPV-negative women were matched by age for comparison of microbiota. The following bacteria were more frequently identified in HPV-positive patients compared to HPV-negative: Eubacterium (68 vs 32%), Actinobacteria (46 vs 33%), Fusobacterium (11 vs 6%) and Bacteroides (20 vs 13%). A comparison between high-risk and low-risk genotypes was performed and differences were found in the detection of Actinobacteria (50 vs 33.33%), Bifidobacterium (50 vs 20.83%) and Enterococcus (50 vs 29.17%).

## Introduction

Human papillomavirus (HPV) infection is the most common sexually transmitted infection worldwide [1]. It is reported that most sexually active individuals are at risk of acquiring this infection at some point of their life and the risk of being infected at least once in a lifetime is approximately 50% [1, 2]. There have been more than 200 genotypes of HPV identified, among them, HPV genotypes 16, 18, 31, 33, 35, 39, 45, 51, 52, 56, 58, 59 and 68 are classified as high-risk or oncogenic types, which cause cervical, anogenital and oropharyngeal neoplasias [1, 3]. High-risk HPV types 16 and 18 are responsible for approximately 70% of all cervical cancers [1].

According to the World Health Organization (WHO) and the Global Cancer Observatory (GLOBOCAN), cervical cancer represents the fourth most common cancer reported in women, accounting for approximately 6.6% of all female cancers [4, 5]. It is the third most frequent neoplasia in women in Peru, with an age adjusted incidence at 34.5 per 100,000 women, higher than the rest of countries in South America [6], probably due to the different barriers for the immunization, detection and treatment in this country [6, 7].

Several studies have shown that the prevalence of HPV is the accumulated number cases of incident and persistent infections combined due to the lack of clearance [1]. More than 90% of new human papillomavirus infections are cleared within 6–24 months from acquisition and more persistent infection is a prerequisite for developing cervical neoplasia [1, 3].

It is still unclear why some HPV infections resolve clinically, and others progress to cervical dysplasia and ultimately, neoplasia. Recently, it has been proposed that women with a specific composition of cervico-vaginal microbiota may be more susceptible to acquire HPV or display a rapid progression to cancerous lesions [8, 9].

The cervico-vaginal microbiota is composed by a variety of microrganisms, usually dominated by different species of *Lactobacillus,* accompanied by other anaerobic bacteria including *Actinobacteria, Fusobacterium, Gardnerella, Atopobium*, *Leptotrichia*, *Sneathia, Prevotella* spp. and other bacterial species [10]. Moreover, fungi have also been identified as part of the vaginal flora, predominantly Candida species [11].

Most of the studies have assessed the impact of the relative abundance of *Lactobacillus* strains present in the cervico-vaginal microbiota; however, the role of other anaerobic bacteria requires further investigation. Therefore, the objective of this study was to characterize the cervico-vaginal microbiota of HPV-positive and HPV-negative asymptomatic Peruvian women, by identifying the presence of 13 representative bacteria genus.

## Main text

### Methods

#### Patients and study design

A case–control study was carried out, using samples and data collected from a previous study performed in *Hospital Regional Docente de Cajamarca*, in northern Peru [12]. Women were screened for the detection of HPV infection from March 2017 to March 2019. Asymptomatic women attending a gynecological outpatient health center, who had a history of at least 1 sexual encounter were studied. Exclusion criteria included: pregnancy, severe gynecological bleeding, previous hysterectomy, previous history of HPV-related disease including cancer, warts and cutaneous manifestations.

A total of 833 women were studied in the context of a screening performed in asymptomatic women from Cajamarca, Peru. Among the 833 samples, 200 samples were positive for HPV and 633 were negative at baseline. For methodological convenience, we selected 100 positive cases and 100 negatives cases matched by age for the comparison of cervico-vaginal microbiota in this study.

#### Sample collection

Samples were collected from the ectocervix and endocervix using a disposable cytobrush, they were stored in a tube containing phosphate buffered saline (pH: 8.6) for preservation. The tubes were maintained at − 4 °C until later processed. All tubes were vortexed and centrifuged to pellet the cells, which were resuspended in 1 mL of phosphate buffered saline. Three aliquots of each sample were stored at − 20 °C until testing.

#### DNA extraction

Samples were kept at − 20 °C until analyzed. DNA extraction from 200 µL of cervico-vaginal samples was performed using the High Pure PCR Viral Nucleic Acid Kit (Roche), according to manufacturer’s instructions. The DNA extracted was stored at − 20 °C until used.

#### PCR for HPV amplification

HPV amplification was carried out using primers and conditions previously described [13]. Amplified products were later analyzed by gel electrophoresis on 2% agarose (FMC, Rockland, ME) containing 7 mg/L of ethidium bromide. The HPV genotypes were classified based on the IARC classification for cancer risk: high, possibly oncogenic and low risk [14].

#### PCR for microbiota amplification

The amplification of 13 representative bacteria genus was performed using primers previously described [15]. The genus of bacteria analyzed were *Actinobacteria, Bacteroides, Bacteroidetes, Bifidobacterium, Clostridium, Enterococcus, Eubacterium, Firmicutes, Fusobacterium, Lactobacillus, Prevotella, Proteobacteria* and *Veilonella*. Conditions of the PCR were an initial incubation of 95 °C for 5 min, followed by 45 cycles at 95 °C for 1 min; 52 °C for 45 s and 72 °C for 1 min; with a final extension at 72 °C for 10 min. The amplified products were later analyzed by gel electrophoresis on 2% agarose (FMC, Rockland, ME).

#### Ethics statement

This study was approved by the Research Ethics Board of the *Hospital Regional de Cajamarca,* Peru. The samples were obtained in the Specialized Oncological Preventive Service of the *Hospital Regional de Cajamarca*, as part of the cervical cancer screening program. The collection of the samples was done with a prior informed consent, which allowed the use of this samples for further studies.

### Results

Demographic characteristics and gyneco-obstetric records of the two groups were compared. HPV-positive patients were aged 35.06 ± 8.86 and HPV-negative were aged 35.72 ± 8.72. The marital status of the patient was also assessed, most of the patients were cohabiting (40 and 46% respectively) in both groups. Also, gynecological and obstetric background information was obtained, as shown in Table 1.

**Table 1:** Demographical and gyneco-obstetric characteristics according to HPV status

Characteristics	HPV + n = 100 (%)	HPV − n = 100 (%)
Age (Me/SD)	35.1 (8.9)	35.7 (8.7)
Marital status (n/%)		
Married Cohabiting Single	15 (15.0) 40 (40.0) 45 (45.0)	25 (25.0) 46 (46.0) 29 (29.0)
Age of first sexual relationship (Me/SD)	18.6 (3.8)	19.1 (4.8)
Number of sexual partners to date (n/%)		
1 2 3 or more	31 (31.0) 42 (42.0) 27 (27.0)	58 (58.0) 26 (26.0) 16 (16.0)
Parity (Me/SD)	1.7 (1.3)	2.1 (1.3)
Abortions (Me/SD)	0.3 (0.6)	0.3 (0.6)
Condom use (n/%)		
Yes No	39 (39.0) 61(61.0)	30 (30.0) 70 (70.0)
History of STD (n/%)		
Yes No	16 (16.0) 84 (84.0)	9 (9.0) 91 (91.0)

Different bacteria genus were compared between the two groups as shown in Table 2. The following bacteria were more frequently identified in HPV-positive patients compared to HPV-negative: *Eubacterium* (68 vs 32%), *Actinobacteria* (46 vs 33%), *Fusobacterium* (11 vs 6%) and *Bacteroides* (20 vs 13%).

**Table 2:** Bacteria genus identified in HPV-positive and HPV-negative asymptomatic women

Bacteria genus	HPV + (n = 100)		HPV − (n = 100)	
	Frequency (n)	Percentage (%)	Frequency (n)	Percentage (%)
*Bacteroides*	20	20.0	13	13.0
*Fusobacterium*	11	11.0	6	6.0
*Actinobacteria*	46	46.0	33	33.0
*Eubacterium*	68	68.0	32	32.0
*Proteobacteria*	69	69.0	68	68.0
*Veillonella*	2	2.0	1	1.0
*Lactobacillus*	98	98.0	93	93.0
*Bifidobacterium*	43	43.0	43	43.0
*Clostridium*	3	3.0	1	1.0
*Firmicutes*	96	96.0	97	97.0
*Bacteroidetes*	38	38.0	41	41.0
*Enterococcus*	45	45.0	44	44.0
*Prevotella*	37	37.0	46	46.0

The variations in microbiota between high-risk (including possibly oncogenic) and low-risk was also evaluated as reported in Table 3. Several bacteria were most frequently identified in high-risk genotypes than in low-risk genotypes, such as, *Actinobacteria* (50 vs 33.33%), *Bifidobacterium* (50 vs 20.83%) and *Enterococcus* (50 vs 29.17%), respectively. Moreover, it can be observed that some bacteria were more frequently identified in high-risk genotypes compared to HPV-negative samples: Bacteroides (21 vs 13%), Actinobacteria (50 vs 33%), Fusobacterium (67.1 vs 32%).

**Table 3:** Bacteria genus identified in high-risk (including possibly oncogenic), low-risk genotypes and HPV- samples

Bacteria genus	High-risk HPV (including possibly oncogenic) (N = 76)		Low-risk HPV (N = 24)		HPV− (n = 100)	
	Frequency (n)	Percentage (%)	Frequency (n)	Percentage (%)	Frequency (n)	Percentage (%)
*Bacteroides*	16	21.0	4	16.7	13	13.0
*Fusobacterium*	9	11.8	2	8.3	6	6.0
*Actinobacteria*	38	50.0	8	33.3	33	33.0
*Eubacterium*	51	67.1	17	70.8	32	32.0
*Proteobacteria*	53	69.7	16	66.7	68	68.0
*Veillonella*	2	2.6	0	0.0	1	1.0
*Lactobacillus*	74	97.4	24	100.0	93	93.0
*Bifidobacterium*	38	50.0	5	20.83	43	43.0
*Clostridium*	1	1.3	2	8.33	1	1.0
*Firmicutes*	72	94.7	24	100.0	97	97.0
*Bacteroidetes*	29	38.1	9	37.5	41	41.0
*Enterococcus*	38	50.0	7	29.2	44	44.0
*Prevotella*	30	39.4	7	28.2	46	46.0

### Discussion

HPV prevalence is a function of both the incidence and the overt persistence of infection [1–3]. Three classes of HPV cofactors determine if HPV infection will persist sufficiently long for precancer to develop, which include viral, host and behavioral variables [16]. Increasing evidence suggests that cervico-vaginal microbiota may be involved in the HPV infection dynamics [9].

It has been proposed that women with a certain composition of cervicovaginal microbiota may be more susceptible to acquire HPV infection, lack clearance of the infection or show a more rapid progression to cervical neoplasia [8]. Most studies regarding HPV infection and microbiota use the term ‘community-state type’ (CST), defined by the relative abundance and diversity of Lactobacillus species. However, the presence of other important bacteria frequently found in the microbiota has been left out.

We found important differences in the frequency of bacteria identified between HPV-positive and HPV-negative Peruvian women, as well as in high-risk and low-risk HPV genotypes. In the present study some important anaerobic bacteria such as, *Eubacterium*, *Actinobacteria*, *Fusobacterium* and *Bacteroides* were more likely to be identified in HPV-positive patients. The main difference was found in the genus *Eubacterium*, being identified in 68% HPV-positive samples and in 32% HPV-negative samples. Similarly, high-risk genotypes showed a higher percentage of Bacteroides, Actinobacteria and Fusobacterium compared to HPV-negative samples. When comparing high-risk and low-risk genotypes, *Actinobacteria*, *Bifidobacterium* and *Enterococcus*, where more likely to be identified in women infected with high risk genotypes. It has been established that these bacteria are usually found in the gut microbiota, however, they are normal transient colonizers of the vaginal microbiota [17].

A variety of studies have found an association between cervico-vaginal microbiota and HPV infection, however most of the results reported in literature differ from ours. Gao et al. [18] performed one of the earliest studies that found differences in the microbiota composition between HPV-positive and HPV-negative healthy women. A greater and more complex bacterial diversity in HPV-positive women was found, also *Gardnerella vaginalis* and *Lactobacillus gasseri* were detected more frequently in women with HPV infection. Similar to our study, Chao et al. [19], reported that some anaerobic bacteria such as *Bacteroides plebeius*, *Acinetobacter lwoffii*, and *Prevotella buccae* were detected significantly more frequently in HPV-positive women.

Also, several studies have examined the dynamics in HPV infection and the role of the cervical microbiota. In contrast with our study, Arokiyaraj et al. [20] reported that *Eubacterium* eligens was associated with HPV clearance. Di Paola et al. [21] found that a CST IV, characterized by *Lactobacillus* depletion and bacterial vaginosis, was a risk factor for HPV persistence. Particularly, the identification of *Atopobium* spp. and *G. vaginalis* could be useful as microbial markers of HPV persistence. In the current study we could not determine whether the presence of the bacteria identified were associated with persistence or clearance of the infection, however, we our findings could lead to future studies to determine the role of these bacteria.

The composition of the cervico-vaginal microbiota is dynamic and responds to different endogenous and exogenous factors [22], such as antibiotic use, systemic hormones, oral contraceptives, frequency of sexual intercourse, socio-economic status, ethnicity, among other factors [17, 22]. In the case of our study, we selected similar groups for comparison, so the cervico-vaginal microbiota in the women studied would not be affected by other exogenous factors. The population studied was homogenous regarding age, place of birth and gyneco-obstetric records.

The human microbiota plays a pivotal role in the development of mucosal immune system and metabolism, and also participates in the wellbeing of the human host [23, 24]. The microbiota is also involved in carcinogenesis, a tight relationship between cancer and microbiome has been identified as the “oncobiome” [25], particularly, colorectal cancer has been the ideal malignancy to study the effects of host-microbe relationship on carcinogenesis. Gut microbiota has an essential role in cancer progression by modulating inflammation and influencing genomic stability of host cells through deregulation of different pathways [26–28]. Different malignancies have been found to be related to disturbances in microbiota such as hepatocellular carcinoma, pancreatic cancer, lung cancer, breast cancer, among others [26–28]. Therefore, the study of the microbiota in HPV infection and cervical cancer pathogenesis is still a topic of interest to better understand the pathophysiology of this disease.

In conclusion, we found significant differences in the frequency of identification of different bacteria genus among HPV-positive and HPV-negative Peruvian women, also between high-risk and low-risk genotypes. A better characterization of the microbiota composition and its impact on HPV infection is still required. The identification of different bacterial species found in the cervico-vaginal microbiota may act as biomarkers for the identification of susceptible women for persistent infections and progression to neoplasia. Also, the microbiota could have a future role as a therapeutic target for the treatment of this disease.

### Limitations

One of the main limitations is that we did not have information regarding antibiotic or probiotic before sampling. Moreover, we did not have the histological evaluation of neoplasia or pre-cancerous lesions in the women studied. Also, because of the nature of our study, we were unable to determine whether the changes in cervicovaginal are induced by HPV infection or the other way around. Moreover, it would be crucial to examine whether the cervical microbiota may have an impact on HPV infection dynamics, such as acquisition, regression, clearance or progression of the infection to neoplasia. Finally, we did not measure the relative abundance of each bacterium, only the frequency of identification.

## Data Availability

Abstraction format used in the study and dataset are available and accessible from the corresponding author upon request in the link: https://figshare.com/s/ed93a1fb23b7e92fabd0.

## Abbreviations

HPV: Human papillomavirus
PCR: Polymerase chain reaction
DNA: Deoxyribonucleic acid
